# Effects of the “ICU Support” team meeting concept on patient-centered and staff-centered outcomes: study protocol for a randomized controlled multicenter study

**DOI:** 10.1186/s13063-023-07754-7

**Published:** 2023-11-26

**Authors:** Marie S. Thommes, Martin Klasen, Johannes Bickenbach, Maria Deja, Kristin Engelhard, Roland Francis, Johannes Gramatté, Gunther Hempel, Kerstin Gmeinwieser, Gabriel Reedy, Daniel Röder, Ines Schroeder, HUMAN-NET CONSORTIUM, Claudia Apel, Susanne Arnold, Silke Barsch, Heiko Baschnegger, Monika Berberich, Christian Bibo, Marc Bodenstein, Christopher Brandl, Arina Bychkova, Enrico Dähnert, Dagmar Ellmer, Georg Engelbart, Nurith Epstein, Martin R. Fischer, Uli Fischer, Sandra Frank, Michelle Fröb, Andreas Güldner, Axel R. Heller, Franziska Jahns, Stefan Kern, Thea Koch, Sylvia Köppen, Susanne Krotsetis, Sophie Lambert, Dorothea Lange, Andrea Lenes, Alexander Mück, Patrick Meybohm, Carla Nau, Sonja Nebl, Katharina Plonien, Katja Preiß, Stephan Prückner, Maximilian Ragaller, Axel Rand, Maria Reden, Marco Reinhardt, Vanessa Rentschler, Bernd Rupprecht, Benedikt Sandmeyer, Michelle Schmidt, Nora Schorscher, Antje Seidel, Philipp Simon, Malte Söhl, Oliver Spring, Sebastian Stehr, Axel S. Steinke, Svenja Teufert, Volker Thieme, Irhad Trozic, Annette Uhlschmied, Steffen Weber-Carstens, Kathrin Wendler, Bernhard Zwißler, Saša Sopka

**Affiliations:** 1 AIXTRA-Competence Center for Training and Patient Safety, Medical Faculty, RWTH Aachen University, Pauwelsstraße 30, Aachen, 52074 Germany; 2 Department of Anesthesiology, University Hospital RWTH Aachen, Pauwelsstraße 30, Aachen, 52074 Germany; 3 Department of Intensive Care Medicine, University Hospital RWTH Aachen, Pauwelsstr. 30, 52074 Aachen, Germany; 4 Department of Anesthesiology and Intensive Care, University Medical Center Schleswig-Holstein, Campus Lübeck, Ratzeburger Allee 160, 23538 Lübeck, Germany; 5 Department of Anesthesiology, University Medical Center Mainz, Langenbeck Str. 1, 55131 Mainz, Germany; 6 Department of Anesthesiology and Intensive Care Medicine CCM/CVK, Charité – Universitätsmedizin Berlin, Augustenburger Platz 1, 13353 Berlin, Germany; 7 Department of Anesthesiology, Friedrich-Alexander-Universität Erlangen-Nürnberg, University Hospital Erlangen, Krankenhausstr. 12, 91054 Erlangen, Germany; 8 Deptartment of Anesthesiology and Intensive Care, Medicine University Hospital Carl Gustav Carus Technische Universität Dresden, Fetscherstr. 74, 01307 Dresden, Germany; 9 Department of Anesthesiology and Intensive Care Medicine, University of Leipzig Medical Center, Liebigstr. 20, 04103 Leipzig, Germany; 10 Clinic of Anesthesiology and Surgical Intensive Care Medicine, Department for Clinical Nursing Science and Development, University Hospital Augsburg, Stenglinstr. 2, 86156 Augsburg, Germany; 11 Faculty of Life Sciences and Medicine, King’s College London, London, UK; 12 Center for Medical Simulation, Boston, USA; 13 Department of Anaesthesiology, Intensive Care, Emergency and Pain Medicine, University Hospital Würzburg, Oberdürrbacher Str. 6, Würzburg, 97080 Germany; 14 Department of Anesthesiology, LMU University Hospital, LMU Munich, Marchioninistr. 15, Munich, 81377 Germany

**Keywords:** ICU staff, Patient safety, Debriefing, Interprofessional communication, Peer support, Teamwork, Randomized controlled trial, Multicenter study

## Abstract

**Background:**

Providing optimal care for critically ill patients is an extremely important but also highly demanding task, both emotionally and physically. The “ICU Support” team meeting concept aims to support intensive care unit (ICU) teams by promoting interprofessional communication, peer support, and patient safety by providing a structure for daily team meetings. This protocol describes a study to explore the effectiveness of “ICU Support” for patient- and staff-centered outcomes.

**Methods:**

ICU Support will be implemented at nine university hospitals located in Germany, following a two-arm randomized parallel group design with an intervention and a control condition and three data collection periods. In the intervention arm, leading ICU personnel (physicians and nurses) will be trained in ICU Support and implement the ICU Support elements into the daily work routine of their units upon completion of data collection period T0 (baseline). In the control arm, ICU Support will not be implemented until the completion of the data collection period T1 (1 month after study start). Until then, the regular daily schedule of the ICU teams will be maintained. The final data collection period (T2) will take place 4 months after the start of the study. Primary outcomes include the number of intensive care complications per patient during their ICU stay during T1 and the sick-related absence of ICU staff during T1. Secondary outcomes include, among others, the average severity of intensive care complications per patient and employee self-reported data regarding their teamwork and patient safety behaviors.

**Discussion:**

The need for healthy and well-trained ICU staff is omnipresent; thus, structured and evidence-based interventions aimed at supporting ICU teams and facilitating patient safety are required. This multicenter study aims to explore the effectiveness of ICU Support for patient- and staff-centered outcomes. The insights derived from this study have the potential to significantly improve ICU patient safety, staff communication, and connectedness and decrease sickness-related expenses and social costs associated with high work demands among ICU staff.

**Trial registration:**

German Clinical Trials Register DRKS00028642. Registered on 4 April 2022.

## Administrative information

**Table:** 

Title {1}	Effects of the “ICU Support” team meeting concept on patient-centered and staff-centered outcomes: study protocol for a randomized controlled multicenter study
Trial registration {2a and 2b}	Registration in the German Clinical Trials Register since 4 April 2022 (DRKS00028642) https://www.drks.de/drks_web/navigate.do?navigationId=trial.HTML&TRIAL_ID=DRKS00028642
Protocol version {3}	22.10.2022, Version 1.0
Funding {4}	No external funding was or will be received for the design of the study, the collection, analysis, and interpretation of data as well as the writing of the manuscript
Author details {5a}	Dr. Marie S. Thommes mathommes@ukaachen.de AIXTRA-Competence Center for Training and Patient Safety, Medical Faculty, RWTH Aachen University, Pauwelsstraße 30, 52074, Aachen, Germany; Department of Anesthesiology, Uniklinik RWTH Aachen, Pauwelsstraße 30, 52074, Aachen, Germany Dr. rer. medic. Martin Klasen mklasen@ukaachen.de AIXTRA-Competence Center for Training and Patient Safety, Medical Faculty, RWTH Aachen University, Pauwelsstraße 30, 52074, Aachen, Germany; Department of Anesthesiology, Uniklinik RWTH Aachen, Pauwelsstraße 30, 52074, Aachen, Germany Prof. Dr. med. Johannes Bickenbach jbickenbach@ukaachen.de Department of Intensive Care Medicine, University Hospital RWTH Aachen, Pauwelsstr. 30, 52,074 Aachen, Germany Prof. Dr. med. Maria Deja maria.deja@uksh.de Department of Anesthesiology and Intensive Care, University Medical Center Schleswig–Holstein, Campus Lübeck, Ratzeburger Allee 160, 23,538 Lübeck, Germany Prof. Dr. med. Kristin Engelhard engelhak@uni-mainz.de Department of Anesthesiology, University Medical Center Mainz, Langenbeck Str. 1, 55131 Mainz Prof. Dr. med. Roland Francis roland.francis@uk-erlangen.de Charité – Universitätsmedizin Berlin, Department of Anesthesiology and Intensive Care Medicine CCM/CVK, Augustenburger Platz 1, 13,353 Berlin, Germany; Friedrich-Alexander-Universität Erlangen-Nürnberg, University Hospital Erlangen, Department of Anesthesiology, Krankenhausstr. 12, 91,054 Erlangen, Germany Dr. med. Johannes Gramatté johannes.gramatte@uniklinikum-dresden.de Deptartment of Anesthesiology and Intensive Care Medicine University Hospital Carl Gustav Carus Technische Universität Dresden, Fetscherstr. 74, 01307 Dresden, Germany Dr. med. Gunther Hempel gunther.hempel@medizin.uni-leipzig.de Department of Anesthesiology and Intensive Care Medicine, University of Leipzig Medical Center, Liebigstr. 20, 04103 Leipzig, Germany M.Sc. Kerstin Gmeinwieser kerstin.gmeinwieser@uk-augsburg.de Clinic of Anesthesiology and Surgical Intensive Care Medicine, Department for Clinical Nursing Science and Development, University Hospital Augsburg, Stenglinstr. 2, 86,156 Augsburg Prof. Dr. Gabriel Reedy gbreedy@harvardmedsim.org Faculty of Life Sciences and Medicine, King’s College London, London, UK; Center for Medical Simulation, Boston, USA Dr. med. Daniel Röder roeder_d@ukw.de Department of Anesthesiology and Intensive Care, Emergency and Pain Medicine, University Hospital Würzburg, Oberdürrbacher Str. 6, 97,080 Würzburg, Germany Dr. med. Ines Schroeder Ines.Schroeder@med.uni-muenchen.de Department of Anesthesiology, LMU University Hospital, LMU Munich, Marchioninistr. 15, 81,377, München, Germany - HUMAN-NET CONSORTIUM PD Dr. med. Saša Sopka ssopka@ukaachen.de AIXTRA-Competence Center for Training and Patient Safety, Medical Faculty, RWTH Aachen University, Pauwelsstraße 30, 52074, Aachen, Germany; Department of Anesthesiology, University Hospital RWTH Aachen, Pauwelsstraße 30, 52074, Aachen, Germany
Name and contact information for the trial sponsor {5b}	University Hospital RWTH Aachen, Pauwelsstr. 30, 52,074 Aachen, Germany
Role of sponsor {5c}	Initiation of the study; organisational responsibility for the study (study coordination; preparation of study documents; monitoring of adherence to study protocol; data collection and analysis)

## Introduction

### Background and rationale {6a}

Intensive care unit (ICU) teams face the important challenge of providing optimal care for critically ill patients. This task is not only complex but also highly demanding, both emotionally and physically. Consequently, ICU professionals report increased levels of stress, exhaustion, and sick-related absence from work [1, 2]. This, in turn, can negatively affect the quality of patient care as, for example, reflected in an increased number of treatment errors and mortality [3, 4]. Medical errors can further elicit work-related stress in healthcare professionals [5]. Such a series of events may lead to a vicious circle that has been further exacerbated by the current COVID-19 pandemic [6]. The need for healthy and well-trained ICU teams has substantially grown ever since. Yet, there still exists a profound gap between the requirements placed on ICU teams to provide optimal patient care (e.g., adapting to fast-paced changes in the workflow, being emotionally resilient) and how work is typically structured and paced at ICUs. For example, improvements of work processes, interprofessional understanding, and psychological support require time and substantial coordinative efforts that are oftentimes missing during stressful shifts.

Previous research suggests that an important means to counteract the impairment of patient safety and demoralization of ICU professionals are *after-action reflections* (also known as *debriefings*) that promote interpersonal contact and collective learning from experiences [7]. If properly conducted, debriefings promote effective teamwork and improve patient care. In their meta-analysis, Tannenbaum and Cerasoli (2013) demonstrated that organizations can improve individual and team performance by 20–25% by applying debriefings. Their findings further suggest that this effectiveness can be boosted through effective facilitation and structure [8]. Additionally, previous research shows that specific debriefing tools not only provide teams with the opportunity to critically reflect on but also foster employees’ emotional health and well-being [7]. Building on this evidence, the meeting concept “ICU Support” was specifically designed for ICU teams to facilitate regular, supportive, and adaptive team interaction and reflection to promote employee well-being and patient care.

ICU Support is based on the “Circle Up” framework [9] which suggests a process for successfully integrating daily team meetings into clinical practice. Following this process, short meetings at the beginning and end of a shift (approx. 5 min each) are supplemented by situational check-ins during work to optimally support employees in a psychologically safe climate. Preliminary data suggests that Circle Up improves work processes and teamwork and promotes interprofessional peer connectedness and well-being [9]. ICU Support builds on guiding principles underlying effective teamwork in healthcare as proposed by Circle Up: [1] supporting the psychological well-being of employees (e.g., through peer support and the opportunity to contribute to solutions) [2] promoting psychological safety by creating an open, appreciative atmosphere in which employees feel safe to share their ideas or ask for help if needed [10] and [3] regularly facilitating the provision of high-quality feedback. Further, ICU Support combines these principles into the promotion of [4] *safety management*—a principle that seems particularly relevant in an ICU context, where treatment errors often lead to disastrous consequences for patients. Safety management refers to the regular team reflections in which critical incidents and treatment errors are collectively identified and analyzed, so that the number of adverse events is minimized [11]. Thus, next to fostering employee well-being through team cohesion and peer support, ICU Support aims to directly improve patient safety at ICUs by installing a system of reporting, discussing, and integrating team members’ insights to collectively learn from errors and to identify solutions.

### Objectives {7}

The aim of the research project presented here is to evaluate the effectiveness of ICU Support regarding (1) ICU patient outcomes and (2) ICU staff outcomes. More concretely, the *primary objective* of this study is to test the hypotheses that the ICU Support intervention (a) reduces the occurrence of complications in intensive care patients and (b) reduces sickness-related absence among intensive care unit staff. *Secondary study objectives* include investigating whether ICU Support improves the perception of ICU staff regarding team collaboration and interaction related to patient safety, as well as exploring how potential effects of the ICU Support intervention develop over time.

### Trial design {8}

The present study is designed as a superiority trial, employing a two-arm randomized parallel group approach and three data collection periods. This design enables the inclusion of both an intervention group and a control group, allowing for a robust comparison of outcomes for demonstrating potential superiority of the intervention compared to the control. Nine university hospitals in Germany will be participating in the study and will be randomly divided into two study arms:

#### Arm 1: intervention

Upon completion of data collection period T0 (baseline), ICU senior personnel (physicians and nurses) will be trained in the ICU Support concept. Simultaneously, other ICU team members will be informed about the content and background of the ICU Support concept by means of a short, standardized information video. With the start of the study, the managers introduce the ICU Support elements into the daily work routine of their units. In this arm, the intervention will be maintained throughout the course of the study and will be supervised and monitored by a staff member from outside the unit.

#### Arm 2: control

In this study arm, ICU Support will not be implemented until the completion of data collection period T1. Until then, the regular daily schedule of the ICU teams will be maintained. However, to avoid a possible confounding of results by a Hawthorne effect (change in performance due to the knowledge of participating in a study) [12], managers and staff in both study arms will be informed about the content and aim of the study after the end of the survey period T0. Upon completion of data collection period T1, ICU managers will be trained in the ICU Support concept, analogous to the intervention arm.

**Fig. 1:**
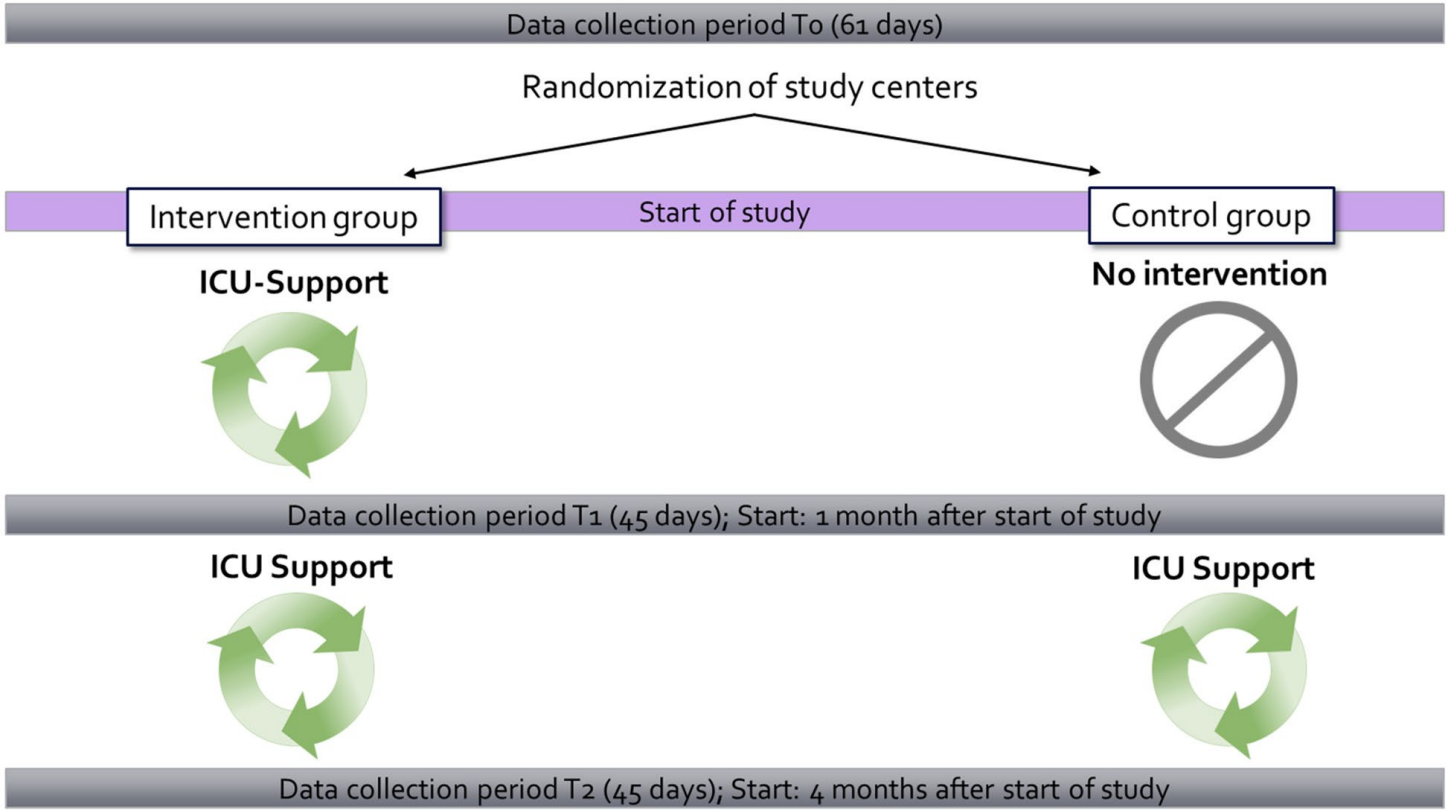
Study design

An overview of the study design is shown in Fig. 1.

Compared to other study designs (e.g., cross-over design), the current study design allows to explore the timing of potential effects of the intervention on patient-centered and staff-centered outcomes, as well as how sustainable such effects are over different time periods. Additionally, assuming that ICU Support positively affects relevant patient and staff outcomes, asking for a termination of the intervention after a certain time period or withholding the intervention from the control group may be ethically questionable and may negatively influence the compliance of ICU staff to participate in the study.

## Methods: participants, interventions, and outcomes

### Study setting {9}

The study will be carried out at nine university hospitals, all of them located in Germany: University Hospital RWTH Aachen, University Hospital Augsburg, Charité – University Medicine Berlin, University Hospital Carl Gustav Carus Dresden, University of Leipzig Medical Center, University Medical Center Schleswig–Holstein Campus Lübeck, University Medical Center Mainz, University Hospital LMU Munich, University Hospital Würzburg. The participating ICUs at the 9 hospitals vary in size, with a range from 51 to 212 employees and an average of 118.4 (SD 49.4) employees.

### Eligibility criteria {10}

#### ICU patients

To be eligible for inclusion in this study, patients have to be admitted to a participating ICU with a minimum length of stay of at least 24 h. Participants are excluded if they are younger than 18 years old, pregnant, or are transferred between study sites during the study period.

#### ICU staff

ICU staff of participating units from study centers involved in patient care (medical service, nursing, therapeutic service including physiotherapists, speech therapists and psychologists, and auxiliary assistants) will be included in the study. Staff will be excluded from study participation if they are younger than 18 years old or if they are involved in activities at multiple study centers during the duration of the study.

### Who will take informed consent? {26a}

Patient-centered outcomes will be anonymously obtained at the unit level at each study site via digital patient records as part of the routine documentation of patient care. Since patients will not receive any study-related intervention directly, they will not be included as study participants but define the patient population through which the quality of care is assessed at each site. Staff-centered outcomes will also be anonymously obtained at the unit level via the Human Resources (HR) department of each clinic and via an online questionnaire using the software SoSci Survey. This software meets the national and international legal requirements for data protection (BDSG, DSG-VO and state laws, ISO-certified). Prior to the participation in the questionnaire, ICU staff will be informed about the study that their participation is voluntary and that non-participation will not result in any negative consequences. Moreover, they will be informed that they can withdraw their participation at any time without providing any reason. Participants can only access the questionnaire after providing online informed consent to participate in the online surveys.

### Additional consent provisions for collection and use of participant data and biological specimens {26b}

All study data will serve only scientific purposes and will be collected and stored anonymously. This study will not involve collecting biological specimens for storage.

## Interventions

### Explanation for the choice of comparators {6b}

The comparison between the number of intensive care complications per patient during the stay in the intensive care unit between study groups was chosen because intensive care complications reflect an important indicator for patient safety at ICUs. This composite measure therefore serves to evaluate the effectiveness of ICU Support on the quality of patient care. Additionally, the comparison between the amount of sick-related leave hours relative to the amount of planned working hours serves to evaluate the effectiveness of ICU Support on sick-related absence of ICU staff; an important indicator of employees’ occupational health and well-being and a factor causing significant payroll costs in hospitals.

### Intervention description {11a}

**Fig. 2:**
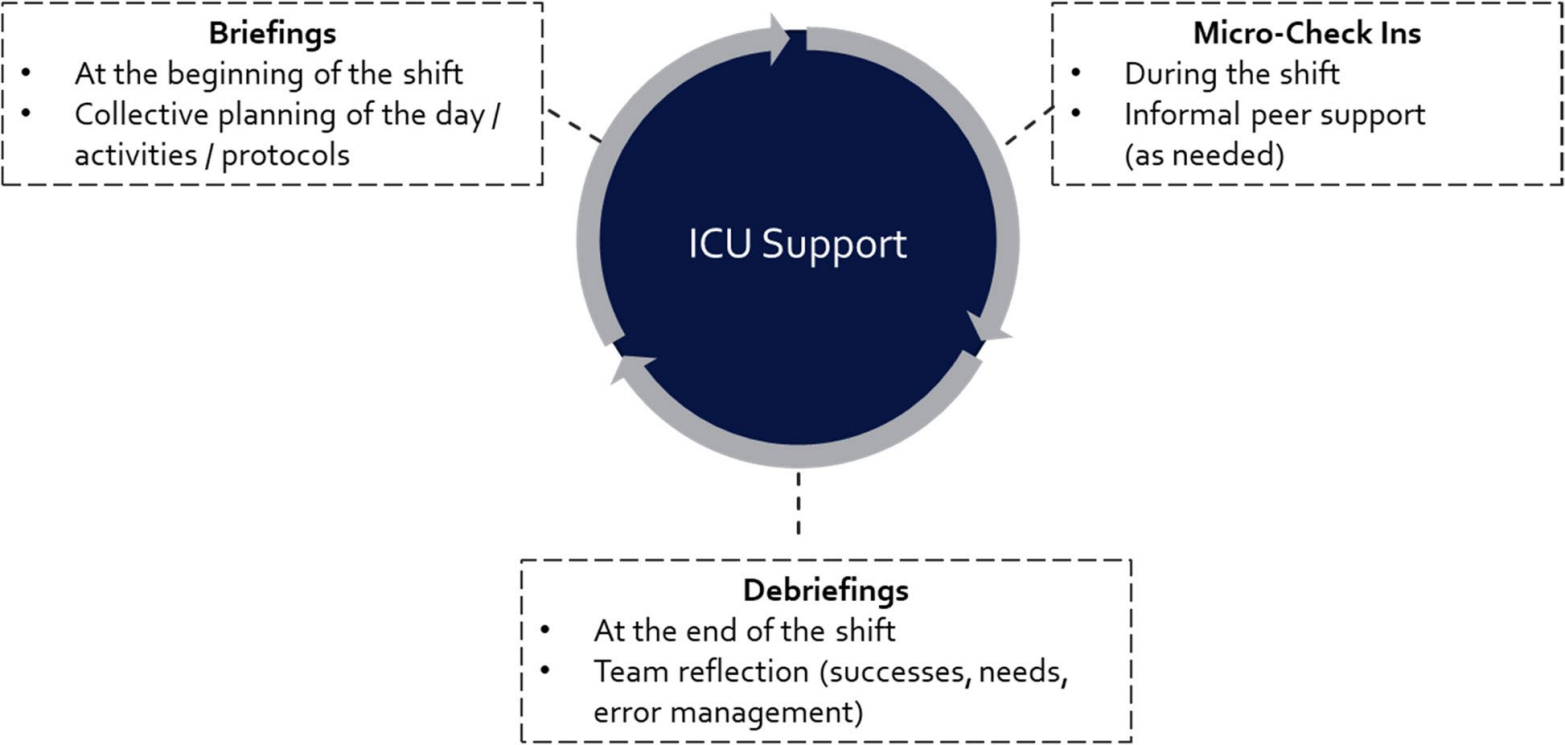
ICU Support. *Note.* Based on Rock et al. (2020) [9], Center for Medical Simulation, NEJM Catalyst (catalyst.nejm.org) © 2020 Massachusetts Medical Society

ICU Support is based on the Circle Up framework [9] and involves three types of main activities that occur on a daily level: (1) *Briefings*, which take place at the beginning of a shift with the whole team or prior to a specific procedure with the team members involved in the activity to plan team-based activities together, [2] *Informal peer check-ins* that take place on an as-needed basis during the shift to offer peer support, and (3) *Debriefings*, which take place at the end of the shift or after a specific procedure to collectively reflect on team successes, to identify critical incidents, to collectively learn from errors, and to establish team needs and learning points to improve work processes and team interaction in the future. Moderators/leaders of the daily briefings or debriefings will be leading personnel from the medical service and nursing that participated in the ICU Support training sessions (see below). Once ICU Support has been fully and regularly implemented, the role of the moderator may be taken up by any team member who feels comfortable in this role and is a trained debriefer. Importantly, the lead of the team meetings should switch between different professions (e.g., physicians, nurses) to ensure high interdisciplinary participation. Figure 2 depicts a visualization of the core elements of ICU Support.

To ensure that ICU Support is successfully implemented at each site, leading professionals of participating ICUs (physicians, nurses) attended a thorough training program prior to the implementation. The training program involved three modules: The first module consisted of a 4-h module in which ICU Support, underlying central team principles (e.g., error management) and their value were introduced. Questions such as “*What is ICU Support? Why is it valuable to us? What makes team meetings successful?*” were collectively discussed and answered in an interactive, face-to-face format. The second module consisted of a 2-h online session in which relevant communication and debriefing techniques were taught to participants and practiced in the form of interactive role-plays. The third module also consisted of a 2-h online session which was specifically dedicated to work out the local implementation strategy of ICU Support at each site (e.g., “*When and where will we conduct the regular team meetings? Who will join? Which practical challenges do we anticipate and how can we solve these?*”).

In addition to the three training modules, the Principal Investigators (PIs) at each site were asked to name 1–2 local contact persons with debriefing experience who were not part of the ICU teams and could locally help operationalize and sustain the ICU Support intervention (e.g., safety-quality staff, psychologists). The local contacts attended the three training modules as described above and received an additional 2-h training on how to monitor and debrief the ICU Support meetings. Prior to the official start of the ICU Support intervention, ICU teams of the participating centers got acquainted with the intervention during a 2-week accommodation phase (a so-called *wash-in* phase). During this phase, ICU team members had the opportunity to try out how to best implement the ICU Support team meetings in their daily clinical practice and adjust their approach if needed (e.g., meeting time, meeting structure, meeting location). Particularly during these 2 weeks, local contacts closely monitored the ICU Support team meetings and provided regular feedback to the ICU teams to assist the effective implementation of the intervention.

**Fig. 3:**
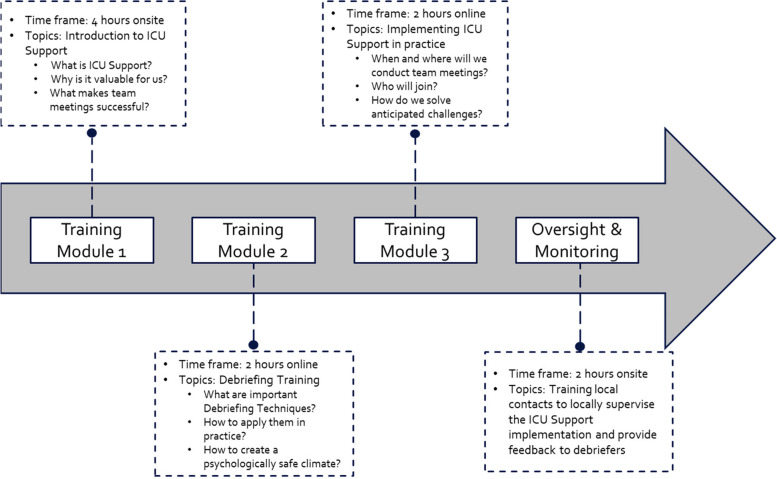
ICU Support implementation process

All training modules were conducted by the same team of debriefing experts and clinicians who were familiar with the ICU Support concept and corresponding operationalization. The training modules were typically distributed over a period of 4 weeks and completed prior of the implementation of ICU Support. Figure 3 shows the implementation process of ICU Support.

### Criteria for discontinuing or modifying allocated interventions {11b}

Allocated interventions will not be discontinued or modified throughout the trial period.

### Strategies to improve adherence to interventions {11c}

Adherence to the intervention will be monitored and documented by the moderators of the ICU Support team meetings by means of a checklist. The checklist will be filled in daily and includes a documentation about whether the Briefing and Debriefing took place on the respective day.

### Relevant concomitant care permitted or prohibited during the trial {11d}

There will be no restrictions on concomitant patient care during the trial.

### Provisions for post-trial care {30}

ICU Support is a structured meeting and support concept for ICU staff developed to promote interprofessional communication, peer support, and patient safety. The intensive care processes themselves are not influenced by the intervention. It is not expected that the implementation of ICU Support will have any negative impact on ICU staff or ICU patients and thus, no specific provisions for post-trial care are required.

### Outcomes {12}

#### Patient-centered primary outcome

Number of intensive care complications per patient during the stay in the intensive care unit during T1. The definition of complications is a priori based on the classification scheme according to Clavien and Dindo [13]. This includes a 5-level graduation of complications by severity. The primary outcome only considers the number of complications, not their severity. Data collection of patient-centered outcomes will be retrospectively obtained from patient records at each participating study center and will be coded by trained study staff.

#### Staff-centered primary outcome

Sick-related absence data for ICU staff will be obtained anonymously at the unit level via the HR department of the participating study center. To make this data comparable across study centers, we assessed sick-related absence as the amount of sick-related leave hours relative to the amount of planned working hours during T1.

#### Patient-centered secondary outcomes

The patient-centered secondary outcomes include the average severity of intensive care complications per patient during their ICU stay in period T1, the number of intensive care complications per patient during their ICU stay in period T2, the average severity of intensive care complications per patient during their ICU stay in period T2.

#### Patient-centered control variables

Control variables include age of patients, gender of patients, previous illnesses, and their total length of stay in the ICU.

#### Staff-centered secondary outcomes

The staff-centered secondary outcomes include sick-related absence during T2, as well as self-reported data on employees’ attitudes towards their work situation that are relevant to patient safety (i.e., dealing with errors, team cooperation, team communication, and management) during T1 and T2. Self-report data will be obtained with the German version of the Safety Attitudes Questionnaire [14] and will consist of 27 items that have been partly modified to be appropriate for the study context.

### Participant timeline {13}

**Fig. 4:**
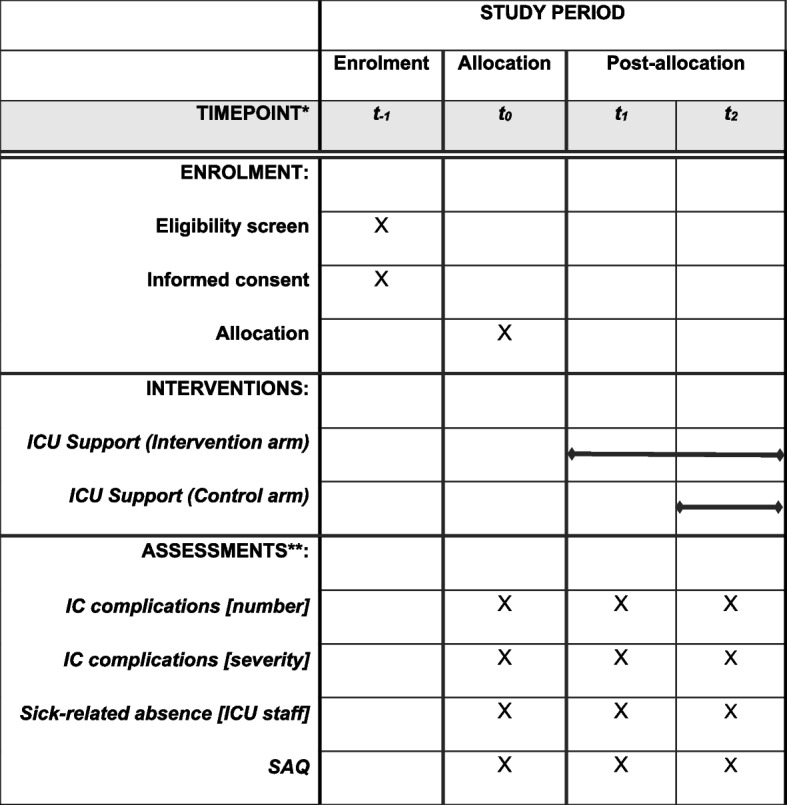
Study timeline. *t_o_ = baseline; t_1_ = 1 month after study start (implementation of ICU Support in the intervention arm); t_2_ = 4 months after study start. ** IC complications [number] = number of intensive care complications according to Clavien-Dindo classification scheme; IC complications [severity] = average severity of intensive care complications according to Clavien-Dindo classification scheme; Sick-related absence [ICU staff] = amount of sick-related leave hours of ICU staff relative to the amount of planned working hours; SAQ = Safety Attitudes Questionnaire (German version)

Participant enrolment, intervention implementation, and assessments will follow a fixed timeline as illustrated in Fig. 4.

### Sample size {14}

Sample size calculations were performed with the statistical software G*Power software version 3.1.9.7 [15] and were based on the respective primary outcome parameter.

#### ICU patients

An analysis of covariance (ANCOVA; fixed effects, main effects, and interactions) with two groups, two measurement times (T0 and T1), and 4 covariates (age, sex, previous illnesses, and length of stay) was scheduled to calculate the appropriate sample size. Assuming a small effect of *f* = 0.10 according to Cohen (1988) [16], an alpha error level of 0.05 and a power of 90%, the total sample size was *N* = 1053. To account for an expected dropout of 10%, a total number of 1110 patients will be targeted.

#### ICU staff

The sample size calculation for the staff-centered outcomes was based on the relative risks (RR) of the two groups (intervention/control) for the measurement times T0 and T1. A small effect of RR = 1.22 according to Olivier et al. (2017) was assumed [17], an average work loss of 2.5% before the intervention, an alpha error level of 0.05, and a power of 90%. This resulted in a total sample of *N* = 37,484 h per time point. Assuming a dropout of 10%, a total number of *N* = 293 employees will be targeted.

### Recruitment {15}

The selection of the centers is based on a previous research project, in which several of the centers participated, as well as via personal contacts. The centers are widely distributed over Germany, located in six different states (North-Rhine Westphalia, Rhineland-Palatinate, Schleswig–Holstein, Bavaria, Saxony, and Berlin). There was no minimum criterion for inclusion, except for the existence of an ICU.

The sample size calculation (please see {14}) was done entirely independently from the center recruitment and based on statistical literature. Since previous literature on similar interventions is scarce (particularly in combination with the outcome variables), it was not possible to derive empirically supported effect size estimates. Therefore, we decided for rather conservative estimates of small effect sizes.

Considering the temporal sequence, sample size planning was performed after the center recruitment. Nonetheless, it was an essential precondition for study initiation since it indicated whether the study was possible at all. As for the patient sample, the duration of the data acquisition periods was defined based on previous data on patient numbers in the respective centers. Data acquisition periods were planned for a duration assuring a sufficient number of patients. As for the employee data, we had no influence on the number of employees; however, we were able to assess the total number of employees (1.066 persons), which was more than three times the required number. There is no expected dropout since the data are collected anonymously via the HR departments, which have complete records about the entire staff.

## Assignment of interventions: allocation

### Sequence generation {16a}

The nine participating study centers were randomly assigned to the intervention or control arm prior to the start of the study, using a web-based randomizer [18]. Following this procedure, four study centers were assigned to the intervention arm and five study centers were assigned to the control arm.

### Concealment mechanism {16b}

The allocation sequence was created during the course of an online video call with representatives of all study centers. The resulting allocation was immediately communicated to the centers; no concealment was used.

### Implementation {16c}

The allocation sequence and accordingly the assignment of the centers to study arms was generated by the study's statistician, who is employed at one of the study centers. Participants (employees and patients) are enrolled by representatives of the centers at their respective locations.

## Assignment of interventions: blinding

### Who will be blinded {17a}

Due to the open nature of the intervention, the staff of the study centers is not blinded to the intervention. Patient-centered data is collected retrospectively and anonymously as part of routine documentation of patient care. According to German legislation, this retrospective use is possible without informed consent; as such, patients will not be informed about the study and thus no blinding is applied.

### Procedure for unblinding if needed {17b}

Not applicable as explained in the previous section.

## Data collection and management

### Plans for assessment and collection of outcomes {18a}

Data collection of the primary and secondary outcome parameters will take place during three time periods (T0, T1, T2). Primary staff-centered and patient-centered outcomes will be obtained via digital records of the participating study center at each site.

#### T0 (baseline)

Data collection period T0 will start 3 months (90 days) before the start of the study and will last for 45 days. Due to administrative aspects at the local study centers, the specific starting point for T0 might vary between study centers, such that within a total time frame of 61 days all data will be collected for 45 consecutive days at each site. Patient-centered data will be collected during the first 45 days with the start of T0. Sickness-related absence (staff-centered primary outcome) will be obtained during the first 30 days with the start of T0. Questionnaire data will be collected during the last 30 days of T0.

#### T1

Data collection period T1 will start 1 month after the start of the study and will last for 45 days. Equivalent to T0, patient-centered data will be collected during the first 45 days of T1, and sickness-related absence will be obtained during the first 30 days. Questionnaire data will be collected during the last 14 days of T1.

#### T2

Data collection period T2 will start 4 months after the start of the study and will last 45 days. All outcome parameters will be obtained as in data collection period T1.

### Plans to promote participant retention and complete follow-up {18b}

To promote participation in the online survey, 20 gift vouchers in the amount of 20€ each per data collection period will be raffled among participating employees. All other data does not rely on study participation of employees or patients; thus, no other measures are planned to promote participant retention.

### Data management {19}

Patient-centered study data will be saved on secured institute drives at each site. The section of the respective drive is only accessible to the persons involved in the study and is protected from unauthorized access by appropriate software as part of the IT support of the respective site. Data will be deleted from the servers after study completion when binding storage regulations have expired. Study staff and the PI(s) at each site will be responsible for evaluating the accuracy, consistency, and completeness of the data obtained at their site before disseminating their data to the lead project partner University Hospital RWTH Aachen, AIXTRA Competency Center for Training and Patient Safety. The complete study data will be stored in a high-security computer database at the location of the lead project partner, ensuring confidentiality in accordance with national data legislation and Good Clinical Practice (GCP). All persons and third parties involved in performing the study analyses (e.g., statisticians) are bound to secrecy and guarantee that no personal data will be coded in the course of the analysis or passed on to third parties or persons not involved in the study.

### Confidentiality {27}

Data collection (both patient- and staff-centered) will be anonymous. Personal data of ICU staff will not be collected and will not be subject to any statistical analyses. Staff councils at each study site were informed about the study and provided their consent. All patient data that serve as the basis for the primary and secondary outcome parameters are collected on a regular basis as part of the documentation of patient care. The data will be collected and stored pseudonymously. The number and severity of intensive care complications will be coded on the basis of the available data by trained study staff.

### Plans for collection, laboratory evaluation, and storage of biological specimens for genetic or molecular analysis in this trial/future use {33}

Not applicable, since no such samples are collected in this study.

## Statistical methods

### Statistical methods for primary and secondary outcomes {20a}

Data will be analyzed with IBM SPSS Statistics software version 25 (IBM Corp., Armonk, NY, USA). To analyze the effect of the ICU Support intervention on the patient-centered primary outcome, an analysis of covariance with time as the within-subject factor (T0 and T1), study group as the between-subject factor (intervention and control), and 4 covariates (age, sex, previous illnesses, and length of stay) will be conducted. Significance will be determined by two-sided 95% confidence intervals.

### Interim analyses {21b}

No interim analyses are intended.

### Methods for additional analyses (e.g., subgroup analyses) {20b}

No additional analyses are intended.

### Methods in analysis to handle protocol non-adherence and any statistical methods to handle missing data {20c}

No such methods are planned or intended. The only data relying on participant's protocol adherence is questionnaire data. All other data are regularly collected by the hospitals during their everyday working routine (either for treatment or for staff management). For these data, no missing data points are expected.

### Plans to give access to the full protocol, participant-level data and statistical code {31c}

Study material and statistical code will be provided by the investigators upon reasonable request.

## Oversight and monitoring

### Composition of the coordinating center and trial steering committee {5d}

The coordinating study center consists of medical doctors, nursing scientists, and psychologists. They organize regular (weekly or biweekly) meetings of the steering committee, which consists of scientific representatives of all participating study centers and makes decisions on details of the study protocol.

### Composition of the data monitoring committee, its role and reporting structure {21a}

A data monitoring committee is not needed for this study, since the intervention does not change any guidelines or methods of patient treatment.

### Adverse event reporting and harms {22}

Considering the nature of the intervention, we have strong confidence that no adverse events or harms will arise. If any adverse events do occur, the local Ethical Committees will be immediately notified.

### Frequency and plans for auditing trial conduct {23}

The Ethical Committee's primary involvement occurs during the study's approval process and will not convene regular meetings for ongoing trial conduct review. Any significant protocol deviations, adverse events, or other ethical concerns that arise during the study will be promptly reported to the local Ethical Committees for their immediate attention and guidance.

### Plans for communicating important protocol amendments to relevant parties (e.g., trial participants, ethical committees) {25}

Protocol amendments will be communicated to the local Ethics Committees by the members of the trial steering committee. Protocols will be updated in the clinical trial registry accordingly.

### Dissemination plans {31a}

Study data will be published in aggregated and anonymous form. Publication of the results is planned in international peer-reviewed journals. Additionally, local study results from single study centers can be used by means of quality improvement.

## Discussion

Previous studies have already addressed the relationship between team factors, team interventions, and aspects of patient safety. For instance, Leroy and colleagues (2012) found a positive relationship of team psychological safety and the amount of treatment error reporting rates [19], and Neily et al. (2010) reported lower surgical mortality after a medical team training for operating room personnel [20]. However, the present findings are either correlational in nature or aim at the improvement of specific technical skills. To our knowledge, the present study is the first interventional study to investigate the influence of a communication concept on patient and staff outcomes.

ICU Support aims to assist ICU teams by providing a structure for regular and short team meetings before, during, and after ICU shifts to facilitate peer support and effective error management (e.g., by collectively identifying adverse events and finding solutions). Thereby, ICU Support can not only help to improve patient safety but also to facilitate effective team interaction and peer support among ICU staff. The insights derived from this study have the potential to significantly improve ICU patient safety, staff communication, and connectedness and decrease sickness-related expenses and social costs associated with high work demands among ICU staff. Although team briefings and debriefings are generally considered important to improve work processes and patient outcomes [8, 20], they are not regularly implemented into daily clinical practice in a structured manner [21]. This study will thus provide important findings on how structured briefings and debriefings can be integrated into daily clinical practice, thereby informing healthcare policy and practice.

For conducting the trial, some important practical aspects must be considered. Most importantly, ICU Support can only be as good as its implementation on the ICU units. Therefore, it is important to identify obstacles in the implementation and find a solution. ICUs are stressful working environments, and it may require some time to find an adequate time and place for the daily activities. Therefore, the study team will actively support the centers in finding an individual solution. Similarly, it is important to inform the teams of the centers about the study background and aims. This is particularly relevant with respect to the aspect of compliance. Experience from an earlier study of the authors suggests that a lack of background information leads to a low participant engagement, which can threaten the success of an intervention seriously [22]. Finally, continuous local support seems inevitable especially in the early phases; thus, local supporters will be educated and will accompany the implementation in each center.

## Trial status

Following the ethical approval of the study protocol, data collection started in March 2022. Currently, the intervention has been implemented in both study groups and data collection is ongoing. We expect the data collection to be completed by December 2022. This is the first version of the study protocol from 21 October 2022.

## Data Availability

The complete study protocol, study material, as well as generated or analyzed datasets will be available from the corresponding author upon reasonable request. Findings resulting from this study -will be published in international peer-reviewed journals. Inconclusive, negative, and positive results will be published.
